# Do functional and biological factors influence the handgrip strength: A systematic review

**DOI:** 10.1177/03080226241293617

**Published:** 2024-11-19

**Authors:** Louise Myles, Fiona Barnett, Nicola Massy-Westropp

**Affiliations:** 1 Occupational Therapy, College of Healthcare Sciences, James Cook University, Townsville, QLD, Australia; 2 Head, Sport and Exercise Science, College of Healthcare Sciences, James Cook University, Townsville, QLD, Australia; 3 School of Health Sciences, University of South Australia, Adelaide, South Australia, Australia

**Keywords:** Norms, handgrip strength, biological and functional factors

## Abstract

**Introduction:**

The measurement of handgrip strength is widely accepted for assessing and evaluating hand function. Age and gender are known factors that correlate directly with the handgrip strength. This review aimed to identify whether other biological and functional factors influence adult handgrip strength and if so, which are the most important.

**Method:**

A systematic review was performed on studies that examined handgrip strength in relation to biological and functional factors including anthropometric characteristics, occupation, hand dominance and ethnicity within a working-aged population.

**Results:**

The search retrieved 19 studies which were critiqued using the McMasters Critical Appraisal Tool. This review concludes an individual’s height, hand length, hand width/palm width, forearm circumference and hand dominance along with their occupation influence handgrip strength in addition to the established categories of age and gender. It is recommended that future research examines how these factors influence handgrip strength to allow for improved interpretation of handgrip strength in comparison to normative data sets.

## Introduction

Grip strength dynamometry is widely accepted as a standard method for assessing handgrip strength (HGS) and in turn upper extremity strength as HGS is widely used to describe overall hand function (Bhat et al., 2021; Bohannon, 2004; Günther et al., 2008). Hand function may be impacted following surgery, neurological conditions or injury (Mathiowetz et al., 1985). Measurement of HGS can evaluate the effectiveness of a rehabilitation intervention, determine a person’s suitability to return to employment or can be used as an objective measure of true effort performance as a component of a functional assessment (Bohannon, 2003; Reuter et al., 2011). Occupational therapy practice is concerned with the relationship between hand function and the performance of activities of daily living (ADL). To engage in ADLs hands are required to perform complex and detailed functions such as grasping and releasing, lifting and carrying and manipulation of objects (Reissner et al., 2019). Various methods have been developed to objectively evaluate hand function considering the performance of everyday activities, with HGS assessment considered a crucial assessment tool in these evaluations (Mitsionis et al., 2009). Hand function is relevant to all occupational therapist clinicians as function can be affected by physical or neurological injury and various health conditions. Therefore, regardless of the area of practice, hand function assessment and evaluation are relevant to all occupational therapists. Research within the past 20 years has expanded the application of HGS assessment from merely a measure of hand function to consider HGS as an essential health indicator with close association to all-cause mortality (Strand et al., 2016). The broad application of HGS as a predictor of health outcomes has become widely accepted within health services; however, this is not the primary focus of HGS testing within the field of occupational therapy. With such significance placed on the interpretation of HGS scores, examining which biological and functional factors influence HGS is hoped to provide context and improved evaluation of HGS scores in relation to an individual’s hand function.

It is widely acknowledged that age and gender are the main factors thought to influence HGS (Agnew and Maas, 1982; Dodds et al., 2016; Massy-Westropp et al., 2011; Mathiowetz et al., 1985). However, more recent studies have supported the consideration of not only the demographic factors of age and gender when comparing HGS to normative values but also functional and biological factors (Anjum et al., 2012; Bohannon et al., 2006; Klum et al., 2012; Leong et al., 2016; Mohammadian et al., 2015; Rostamzadeh et al., 2020a). For the purpose of this review, examples of functional factors include a person’s occupation and hand dominance, whereas biological factors include height, weight, and various anthropometric values. It is believed that the consideration of other predictive factors would provide improved evaluation of an individual’s HGS including comparison to normative data sets which are used to interpret HGS scores and to report on HGS findings. A study by De Andrade Fernandes et al. (2014) cautioned that the inclusion of additional biological and functional factors in an equation to predict HGS, particularly the inclusion of weight, height and body mass index (BMI) may result in inaccurate assessment. This is because muscular strength may be affected by various other factors in addition to those mentioned. Although numerous studies have investigated various functional and biological factors in relation to HGS, limited studies follow the complete standardised HGS testing protocol as outlined by the American Society of Hand Therapists (ASHT; MacDermid et al., 2015). Furthermore, limited consensus has been reached regarding which biological and functional factors provide the strongest prediction of HGS.

Following the assessment of HGS, evaluation and interpretation of these scores occurs in various forms including reference to normative data. Clinical evaluation of HGS test scores does not always involve comparison to normative data sets. Often, the contralateral or uninjured upper limb is used to compare and to gauge expected strength (Günther et al., 2008). However, utilising normative data sets to evaluate an individual’s ability in comparison to the relative population is essential when making informed decisions (Innes, 1999). Additionally, in order to identify HGS impairments, normative data sets are required to allow for comparison to a normal population (Wang et al., 2018).

Due to the significance and the variety of applications for HGS testing as an assessment tool for health professionals across a variety of practice settings and for numerous purposes within the profession of occupational therapy, it is critical to establish whether biological or functional factors influence HGS. The findings of this review will assist in determining whether functional and biological factors should be considered when interpreting and evaluating a client’s HGS scores in comparison to normative data sets. Specifically, the aim of this review was to identify whether various biological and functional factors influence HGS normative data for working adults and if so, which are most significant.

## Method

The following inclusion and exclusion criteria were developed to ensure the relevancy of the articles reviewed. The date range for inclusion was limited to 2010–2023 due to the volume of publications that matched the key word search as numerous studies have examined HGS and biological factors. The date range was also restricted to ensure the most recent research available was included. Only studies published in English were included. All studies included within the review utilised a cross-sectional study design and described normative HGS data organised by gender and age as a minimum. When considering the adult population to allow for the consideration of the influence of occupation studies which examined a broad adult population incorporating older aged adults were included, with some studies examining the influence of occupation specifically. Studies that exclusively examined older aged populations (beyond the working age) were excluded to narrow the focus toward working aged adults.

Key inclusion criteria included the use of a Jamar dynamometer for HGS testing and the application of the ASHT testing protocol within the study’s methodology. Numerous studies have examined HGS and potential influencing factors; however, limited investigators adhered to all aspects of the testing protocol as outlined by the ASHT. Due to the wide range of HGS protocols identified in the research findings, inclusion of this methodological criteria significantly limited the number of studies included within the review and allowed for a reliable and consistent comparison across the included studies. The ASHT testing protocol requires participants to begin the assessment sitting upright with both the hips and knees in 90° flexion with feet flat on the floor, testing arm at sides, not touching the body, elbow flexed at 90°, forearm in neutral position, wrist slightly extended between 0° and 30° and ulnar deviation between 0° and 15°, and the non-testing arm relaxed at side. Three trials of each hand are taken by alternating between right and left hands and the average of the three trials is the recorded score (MacDermid et al., 2015).

Research studies which met the following inclusion criteria were included for review:

- Published within 2010–2023
- Published in the English language
- Adult population
- Working age population to allow for the consideration of the influence of occupation
- Healthy participants
- Jamar dynamometer for handgrip strength testing
- ASHT testing protocol (including three alternating trials on each hand, with the average score recorded)
- Focus on the influence of the identified biological and functional factors on HGS

Research articles were excluded from the review based on the following criteria:

- Non-English language studies
- Paediatric and older adult studies (beyond working age)
- Studies focusing on the impact of HGS on overall health/fitness/physical performance/other health-related assessments
- Secondary research (systematic reviews/meta analyses)
- Use of non-Jamar dynamometer for HGS testing
- Studies which did not follow the ASHT testing protocol (including three alternating trials on each hand, with the average score recorded)

## Information searches and sources

The literature search strategy was developed by one author (LM) and an independent research librarian. A literature search was conducted in October 2023 for research studies examining HGS and the identified functional and biological factors that affect HGS.

Free-text key words including exploring all terms under each subject heading and MeSH terms were used in combination (using Boolean operators) to systematically search the following databases: Medline, CINAHL, Scopus and InformIT. Specific keywords and phrases used included ‘hand strength’, ‘grip strength’, ‘handgrip strength’, ‘normal range*’, ‘reference values’, ‘hand dominance’, ‘ambidexter*’, ‘anthropometr*’, ‘population’, ‘occupation’, ‘employment’ ‘vocation’. Examples of specific MeSH terms included ‘“hand strength AND reference values”’. A hand search using reference lists from the retrieved articles was also undertaken to elicit any additional articles that met the search criteria these articles were then reviewed in regard to the inclusion and exclusion criteria.

## Data collection and integration process

Each study retrieved from the four databases and the hand search of reference lists was evaluated by one reviewer for inclusion in the review at the title, abstract and full article stages with a second reviewer performing informal sample checks periodically to ensure rigour and minimise selection bias. Full text articles were reviewed by two researchers (LM and FB) to confirm their suitability for inclusion in the review and ensure consistency and rigour. Data items were extracted using the following headings: Reference, sample size, study design, study purpose, variables measured, methodology and results (Table 1) and results of the collated data were integrated narratively.

**Table 1.** Details of handgrip strength protocols and variables measured in the included studies.

Reference	McMaster score	Sample	Design	Purpose	Variables measure	Methodology	Results
Angst et al. (2010)	10	Convenience sample from the community between aged 18–96 years *m* = 496 *f* = 482	Cross-sectional – one point of data collection	To quantify the predictive power of easily assessable demographic and/or anatomical factors such as sex, age, occupational demands on the hand, body height, and body weight on grip and pinch strength. The second aim was to predict grip and pinch strength by a regression model of these factors.	• Age • Gender • Dominant hand was also determined by a standardised questionnaire • Height • Weight • Demands on the hand due to occupational activity (classified into six categories: beyond sedentary, sedentary, light, medium, heavy, very heavy) as set out in the directory of occupational titles.	• Jamar Dynamometer (second handle position) • American Society of Hand Therapists testing procedure • Average of three measurements	• Height had the highest bivariate predictive power (0.680), followed by sex (0.635), age (0.460), weight (0.460) and occupation (0.377) • Sex was the strongest multivariate term • The overall predictive power of these cofactors combined was very high.
Anjum et al. (2012)	8	Convenience sample from hospital staff also individuals from social gatherings, religious congregations aged 20–70 year 105 Asians (*m* = 56, *f* = 49) and 103 Europeans (*m* = 52, *f* = 51).	Cross-sectional – one point of data collection	To assess normal grip and pinch strengths in Asian participants and to compare the corresponding values with the European population.	• Age • Gender • Hand dominance • Height • Weight	• Jamar Dynamometer (second handle position) • American Society of Hand Therapists testing procedure • Average of three measurements	• Europeans showed higher HGS than Asians • Mean HGS was higher on the right side (dominant) than the left in Europeans • Grip strength significantly related to the weight (*p* value <0.01, 95% CI 0.35–0.64) and height (*p* value <0.01, 95% CI 0.65–0.82) in Europeans; but not to the body mass index (*p* value <0.53, 95% CI 0.13–0.25) in both Asian and European groups.
Bhat et al. (2021)	9	Convenience sample of 210 (*m* = 105, *f* = 105) university students aged 18–35 years. 30 students from each of the eight identified ethnic communities (African, Iranian, Chinese, Dutch, Polish, Indian, Malays).	Cross-sectional – one point of data collection	To evaluate whether hand dynamometry varies among young adults based on gender and various ethnicities, which correlates with their grip and pinch strength.	• Age • Gender • Hand dominance • Height • Weight • Arm length • Forearm length • Forearm circumference • Wrist circumference • Hand length • Hand circumference • Maximum fiver finger span • Digit length for all five digits	• Jamar Dynamometer (Second handle position) • American Society of Hand Therapists testing procedure • Average of three measurements	• Differences among various ethnic groups in regard to the anthropometric values and GS (*p* = 0.000) • HGS was maximum in Dutch males and Malay females • Out of the 13 anthropometric measurements, 12 parameters correlated with grip strength except for arm circumference which showed no correlation (*p* = 0.295).
De Andrade Fernandes et al. (2013)	9	Randomly selected sample throughout cities in Brazil *m* = 1279 aged 14–59 years.	Cross-sectional – one point of data collection	To verify the associations of the dominant hand values with weight, height and BMI To gather data concerning normal HGS in men from the Zonada Mata region of the state of Minas Gerais, Brazil.	• Age • Gender • Dominant handed - defined as the hand favoured for performing daily activities, such as writing, eating and handling heavy objects • Height • Weight	• Jamar Dynamometer (Second handle position) • American Society of Hand Therapists testing procedure • Average of three measurements • 1 minute rest breaks between trials	• Weak positive association between height and grip strength of the dominant hand (Spearman’s *r* = 0.28, *p* < 0.01) • Moderate positive association between the dominant HGS and body weight (Spearman’s *r* = 0.316, *p* < 0.01) • Weak positive association between BMI and dominant HGS (Spearman’s *r* = 0.19, *p* < 0.01)
Eidson et al. (2017)	8	Convenience sample of 159 participants (*m* = 36, *f* = 114) from Birmingham, Alabama, US aged 19–34 years	Cross-sectional – one point of data collection	To investigate anthropometric measurements associated with maximal hand grip strength of healthy adults ages 19–34 years	• Age • Gender • Dominant hand – identified as which hand they write with • Height • Weight • Forearm length • Forearm circumference • Hand length • Hand width	• Jamar Dynamometer (Second handle position) • American Society of Hand Therapists testing procedure • Average of three measurements • 10 second rest breaks between trials	• Gender and hand width significantly associated with maximal HGS (*p* value <0.001) • Hand width or forearm circumference are the best hand anthropometric measures to estimate maximal HGS
Hatem et al. (2016)	9	Convenience Random sample of 1029 participants (*m* = 524, *f* = 505) from urban, suburban and rural areas from a wide variety of settings 20–85 years	Cross-sectional – one point of data collection	To identify the relationship between Age, Anthropometric measurements as height and weight and Hand Grip Strength in both right and left hands.	• Age • Gender • Hand dominance • Height • Weight	• Jamar Dynamometer (Second handle position) • American Society of Hand Therapists testing procedure • Average of three measurements • 1 minute rest breaks between trials (alternating hands)	• Age inversely correlated with Grip strength for both right and left hands in both females and males (*p* value <0.0001) • Height and weight showed significant correlation with Grip strength for both right and left hands in both females and males (*p* value <0.0001), except that the weight did not correlate with female right hand grip strength
Klum et al. (2012)	9	Convenience sample of 750 participants (*m* = 387, *f* = 363) aged 18–65 years	Cross-sectional – one point of data collection	To investigate the predictive power of the parameters age, gender, body height, body weight, BMI, occupational manual strain DASH score and ROM on grip strength A second goal was to develop models that enable the prediction of grip strength using multiple regression models.	• Age • Gender • Dominant hand • Occupational manual strain • Height • Weight • AROM wrist extension/flexion and ulnar/radial deviation • DASH questionnaire	• Jamar Dynamometer (Second handle position) • American Society of Hand Therapists testing procedure • Alternating hands between trials • Average of three measurements	• Gender was the most important parameter in predicting hand strength (*p* value <0.0001) • Highly significant correlation between hand strength with body height, body weight and BMI (*p* value <0.0001) • Highly significant negative influence of age (*p* value <0.0001) • The extension and flexion of the wrist correlated positively with grip strength (*p* value <0.0001) • Occupational manual strain had no significant influence on hand strength (*p* value 0.50)
Langer et al. (2022)	10	Convenience sample of 637 (*m* = 334, *f* = 293) community based adults aged 18–70+	Cross-sectional – one point of data collection	To establish normative data for grip strength for the adult population in Israel. The second objective was to compare the results of this study to international normative data.	• Age • Gender • Dominant hand • Type of work (high or low manual strain)	• Jamar Dynamometer (second handle position) • American Society of Hand Therapists testing procedure • Alternating hands between trials • Average of three measurements	• HGS among men exceeded HGS in women • Progressive decline in HGS with increasing age • For both men and women, the dominant hand was stronger than the non-dominant • Results of a Welch’s *t*-test showed a medium to large effect for type of work with high manual strain workers having stronger HGS.
Massy-Westropp et al. (2011)	10	Random sample of 2629 (*m* = 1314, *f* = 1315) aged 20 years and over living in Adelaide Australia	Cross- sectional Stage 1 – stratified random sampling) Stage 2 attend – clinic for assessment	To describe normative data for handgrip strength in a community-based Australian population. To investigate the relationship between BMI and handgrip strength, and to compare with international handgrip strength norms.	• Age • Gender • Dominant hand • Height • Weight • BMI	• Jamar Dynamometer (second handle position) • American Society of Hand Therapists testing procedure • Average of three measurements	• Analysis by Pearson *r* correlation, with a significance level of 0.05 • A very weak positive relationship between higher BMI and right HGS for the youngest and oldest adults • For young adults and those in their fourth, fifth and sixth decade, a higher BMI was inversely related to HGS.
Mohammadian et al. (2016)	10	Stratified random sampling method from the adult Iranian population aged 20–107 years *m* = 526 *f* = 482	Cross-sectional – one point of data collection	To investigate the correlation of anthropometric and demographic factors with hand strength as well as to develop regression models for grip and three types of pinch strengths including Tip, Key and Palmar in Iranian adult population.	• Age • Gender • Ethnicity • Dominant hand • Physical demands levels • Height • Weight • Hand length • Hand width • Mid-arm circumference • Forearm circumference • Hand span	• Jamar Dynamometer (Second handle position) • American Society of Hand Therapists testing procedure • Average of three measurements • 1 minute rest breaks between trials	• HGS of females significantly lower than those of the males (*p* value <0.0001) • Inverse and significant correlation between age and HGS (*p* value < 0.0001) • Positive and significant correlation between HGS and anthropometric dimensions • Highest correlation of HGS with height, hand span, forearm circumference and hand length dimensions • No significant difference between physical demand levels and HGS for both genders.
Moy et al. (2015)	9	Multistage sampling of households within five randomly selected districts in rural Malaysia *m* = 927 *f* = 1142 aged 30 years and older	Cross-sectional – one point of data collection	To determine the predictors of handgrip strength among adults of a rural community in Malaysia	• Gender • Age • Dominant hand • Height • Weight • BMI	• Jamar Dynamometer (Second handle position) • American Society of Hand Therapists testing procedure • Three measurements on each hand • 15 second rest break between trials • Highest of three measurements chosen for analysis	• Males had higher HGS compared with females (*p* value <0.001) • HGS declined as age increased for both genders (*p* value <0.05) • Those with medical conditions such as diabetes mellitus, hypertension and high cholesterol had significantly lower HGS (*p* value <0.01) • Positive and significant correlations between HGS and height (*p* value <0.001), weight (*p* value <0.001) and musculoskeletal score (*p* value <0.001) among males only • In the multivariate model for males, age, height, job groups and diabetes significantly predicted HGS • Dominant HGS significantly higher than non-dominant • Compared with the population in the West, participants had significantly lower HGS.
Rostamzadeh et al. (2019)	10	Convenience sample of 418 (*m* = 220, *f* = 198) office employees aged from 20 to 60 years.	Cross-sectional – one point of data collection	To establish GS norms of Iranian office workers stratified by gender, age-group and hand dominancy. To review the correlation between GS and different demographic and anthropometric variables To investigate the predictors of GS among the study population and to develop the appropriate predictive equations.	• Age • Gender • Dominant hand – identified as which hand they write with • Height • Weight • BMI • Hand length • Palm width • Palm length • Forearm length • Wrist circumference • Forearm circumference • (all measured as per NASA anthropometric source book)	• Jamar Dynamometer (Second handle position) • American Society of Hand Therapists testing procedure • Average of three measurements • 1 minute rest breaks between trials	• There was a significant correlation between HGS and all measured variables, except BMI; suggesting that HGS increases as height, weight, hand length, palm width, palm length, forearm length, wrist circumferences and forearm circumferences of an office worker increase • HGS had the highest correlation (*p* < 0.01) with palm width followed by palm length and hand length, respectively.
Rostamzadeh et al. (2020a)	11	Stratified random sample from a public university in Iran 1740 male workers aged 20–64 years Two occupational groups: light manual workers and office workers	Cross-sectional – 1 point of data collection	To compare maximum HGS between light manual workers and office employees and investigate if the expected differences are related to anthropometric dimensions of the workers’ forearms and hands	• Age • Gender • Dominant hand – identified as which hand they write with • Height • Weight • BMI • Hand length • Palm length • Handbreadth • Wrist circumference • Forearm length • Forearm circumference	• Jamar Dynamometer (Second handle position) • American Society of Hand Therapists testing procedure • Average of three measurements • 1 minute rest breaks between trials	• Maximum HGS of light manual workers was significantly higher then office workers for both hands • Dominant HGS was stronger on average than non-dominant for all workers • HGS increased until 35–39 years then gradually decreased for both work groups • Hand breadth and forearm circumference were significantly different between the two groups of workers with light manual workers having greater hand breadth and forearm circumference in both upper limbs • Weight, height and BMI highly correlated with HGS • Hand breadth and forearm circumference highest correlation with HGS for both groups of workers.
Rostamzadeh et al. (2020b)	10	Quasi random sample from shopping centres and malls, service centres and public areas to ensure a wide variety of occupational, ethnic and socioeconomic backgrounds. 4282 Iranians (*m* = 2167, *f* = 2115). two occupational groups: MW and NMW	Cross-sectional – 1 point of data collection	To create normative data for GS in Iranian healthy population stratified by age, gender and hand side To compare GS of Iranian population with consolidated and international norms To investigate individual predictors of GS among studied demographic and anthropometric parameters To develop prediction equations for GS in Iran.	• Age • Gender • Dominant hand – identified as which hand they write with • Type of work; MW or NMW • Height • Weight • BMI • Hand length • Palm width • Palm length • Forearm length • Wrist circumference • Forearm circumference • (all measured as per NASA anthropometric source book)	• Jamar Dynamometer (Second handle position) • American Society of Hand Therapists testing procedure • Average of three measurements • 1 minute rest breaks between trials	• Hand dominance had a significant effect on HGS (*p* < 0.001). Dominant hand stronger than non-dominant hand by about 10% and 11% for males and females, respectively • MWs were stronger HGS compared to NMWs, on both sides (*p* < 0.001). Among NMWs, students had a weaker grip than non-students • HGS increases with increasing weight, height, BMI, hand length, palm length, palm width, forearm length, wrist circumference and forearm circumference • Palm width has the highest correlation with HGS in both genders (*p* < 0.01) for dominant and non-dominant hands.
Saremi and Rostamzadeh (2019)	10	Stratified random sampling methods from difference organisations and companies. *m* = 1740 aged from 20 to 64 years Two occupational groups: LMW tasks (905) and office/clerical workers (835)	Cross-sectional – one point of data collection	To investigate whether light manual workers have higher GS compared to office/clerical employees as non-manual workers To determine anthropometric differences between the two occupational groups. and To determine demographic and anthropometric factors related to GS in each occupational group.	• Age • Gender • Dominant hand – identified as which hand they write with • Work category • Height • Weight • BMI • Hand length • Palm width • Palm length • Forearm length • Wrist circumference • Forearm circumference • (all measured as per NASA anthropometric source book)	• Jamar Dynamometer (Second handle position) • American Society of Hand Therapists testing procedure • Three consecutive measurements on each hand • 1 minute rest break between trials	• HGS in LMW significantly higher than office/clerical employees • Palm width and forearm circumference significantly different between the two occupational groups • LMW had greater palm width and forearm circumference than office/clerical employees • All demographic and anthropometric factors significantly correlated with HGS • Palm width most significant correlation with HGS for LMW and office/clerical employees • Forearm circumference correlated to HGS for dominant and non-dominant of both occupational groups.
Saremi et al. (2021)	11	Certified dentists who specialised in one of three specialities of maxillofacial surgery, endodontics or paediatric dentistry Purposive sample of 720 dental specialists *m* = 330 *f* = 390	Cross-sectional – one point of data collection	To investigate the relationship between dentists’ hand functionality and dental speciality, socio-demographic factors and hand-forearm anthropometrics dimensions	• Age • Gender • Dominant hand – identified by classifying the non-dominant hand as the hand which holds the mirror during treatment • Dental speciality • Height • Weight • BMI • Hand length • Palm length • Palm width • Wrist circumference • Forearm circumference • Forearm length • Clinical experience	• Jamar Dynamometer (Second handle position) • American Society of Hand Therapists testing procedure • Three consecutive measurements on each hand • 1 minute rest break between trials	• Average anthropometric measures higher in males than females • Height and weight significantly correlated to HGS • Hand length and forearm strongly correlated with HGS • Palm dimensions (length and width) correlated with HGS • Wrist and forearm circumferences moderately correlated with HGS • Significant effect for gender and age with HGS higher in males than females for all ages • Male and female maxillofacial surgeons had higher mean HGS and forearm circumference than the other specialists • Clinical experience negatively correlated to HGS for all specialties, suggesting HGS decreased with increasing seniority in dental work.
Shim et al. (2013)	9	Convenience sample of patients visiting health institution *m* = 137 *f* = 199 aged 13–77 years	Cross-sectional – one point of data collection	To establish the normal values of grip and pinch strength among the healthy Korean population and to identify any dependent variables affecting grip and pinch strength.	• Age • Gender • Dominant hand • Height • Weight • Hand width • Hand length • Forearm length • Forearm circumference	• Jamar Dynamometer (Second handle position) • American Society of Hand Therapists testing procedure • Three consecutive measurements on each hand • 1 minute rest break between trials	• All mean strength measurements significantly greater in males than in females (*p* value <0.01) • HGS increased into young adulthood and then declined among the geriatric population • Hand dominance had no significant correlation with measured variables, however dominant hand had greater HGS • All metrics but the forearm lengths found to correlate with male HGS (*r* = 0.4–0.5) • No significant correlations found between HGS and all metrics for females
Spruit et al. (2013)	10	*m* = 224,852 *f* = 224,830 white ethnic background aged 39–73 years as part of the United Kingdom biobank prospective epidemiological study	Cross-sectional – one point of data collection	To develop normative values for right and left handgrip strength after stratification for confounders like gender, age, and height. To develop new normative values for handgrip strength, after stratification for sex, age, and height using individuals from the large UK Biobank dataset without chronic conditions.	• Age • Gender • Height	• Jamar Dynamometer (Second handle position) • American Society of Hand Therapists testing procedure • Three consecutive measurements on each hand	• Men were stronger than women (*p* value <0.001) • A weak inverse correlation was found between HGS and age (*p* value <0.01) • A strong positive correlation was found between HGS and height (*p* value <0.01) • Men, younger individual, and taller individuals had higher HGS compared with women, older individuals and shorter individuals
Wang et al. (2018)	9	Community dwelling and non-institutionalised United States residents aged 18–85 years as part of the normative phase of the NIH Toolbox project *m* = 449 *f* = 783	Prospective cohort study	To provide population-based grip strength reference values and equations for United States residents 18–85 years. Normative data will enable comparison of grip strength values in individuals with or without impairments to the reference values and allow clinicians to provide feedback during the rehabilitation process	• Age • Gender • Dominant hand • Height • Weight • BMI • Ethnicity (including language spoken) • Education	• Jamar Dynamometer (Second handle position) • American Society of Hand Therapists testing procedure • Single maximal trial of 3–4 seconds	• HGS differed significantly by sex (men stronger than women), hand dominance (dominant side stronger) and age (younger adults stronger than older adults) (*p* value <0.001) • Stronger correlation between HGS and height (*r* = 0.61) than the correlation between grip strength and BMI (*p* value <0.045) • Three variables identified (weight, height and aged cubed) for inclusion in reference equations • Participants with a high school diploma or higher degree were stronger than participants who did not finish secondary education

Reference	McMaster score	Sample	Design	Purpose	Variables measure	Methodology
Klum et al. (2012)	Convenience sample of 750 participants (*m* = 387 *f* = 363) aged 18–65 years	Cross-sectional – one point of data collection	To investigate the predictive power of the parameters age, gender, body height, body weight, BMI, occupational manual strain DASH score and ROM on grip strength A second goal was to develop models that enable the prediction of grip strength using multiple regression models.	• Age • Gender • Dominant hand • Occupational manual strain • Height • Weight • AROM wrist extension/flexion and ulnar/radial deviation • DASH questionnaire	• Jamar Dynamometer (second handle position) • American Society of Hand Therapists testing procedure • Alternating hands between trials • Average of three measurements	• Gender was the most important parameter in predicting hand strength (*p* value <0.0001) • Highly significant correlation between hand strength with body height, body weight and BMI (*p* value <0.0001) • Highly significant negative influence of age (*p* value <0.0001) • The extension and flexion of the wrist correlated positively with grip strength (*p* value <0.0001) • Occupational manual strain had no significant influence on hand strength (*p* value 0.50)
Langer et al. (2022)	Convenience sample of 637 (*m* = 334, *f* = 293) community based adults aged 18–70+	Cross-sectional – one point of data collection	To establish normative data for grip strength for the adult population in Israel. The second objective was to compare the results of this study to international normative data.	• Age • Gender • Dominant hand • Type of work (high or low manual strain)	• Jamar Dynamometer (Second handle position) • American Society of Hand Therapists testing procedure • Alternating hands between trials • Average of three measurements	• HGS among men exceeded HGS in women • Progressive decline in HGS with increasing age • For both men and women, the dominant hand was stronger than the non-dominant • Results of a Welch’s *t*-test showed a medium to large effect for type of work with high manual strain workers having stronger HGS
Massy-Westropp et al. (2011)	Random sample of 2629 (*m* = 1314, *f* = 1315) aged 20 years and over living in Adelaide Australia	Cross-sectional Stage 1 – stratified random sampling) Stage 2 attend – clinic for assessment	To describe normative data for handgrip strength in a community-based Australian population. To investigate the relationship between BMI and handgrip strength, and to compare with international handgrip strength norms.	• Age • Gender • Dominant hand • Height • Weight • BMI	• Jamar Dynamometer (Second handle position) • American Society of Hand Therapists testing procedure • Average of three measurements	• Analysis by Pearson *r* correlation, with a significance level of 0.05 • A very weak positive relationship between higher BMI and right HGS for the youngest and oldest adults • For young adults and those in their fourth, fifth and sixth decade, a higher BMI was inversely related to HGS
Mohammadian et al. (2016)	Stratified random sampling method from the adult Iranian population aged 20–107 years *m* = 526 *f* = 482	Cross-sectional – one point of data collection	To investigate the correlation of anthropometric and demographic factors with hand strength as well as to develop regression models for grip and three types of pinch strengths including Tip, Key and Palmar in Iranian adult population.	• Age • Gender • Ethnicity • Dominant hand • Physical demands levels • Height • Weight • Hand length • Hand width • Mid-arm circumference • Forearm circumference • Hand span	• Jamar Dynamometer (Second handle position) • American Society of Hand Therapists testing procedure • Average of three measurements • 1 minute rest breaks between trials	• HGS of females significantly lower than those of the males (*p* value <0.0001) • Inverse and significant correlation between age and HGS (*p* value <0.0001) • Positive and significant correlation between HGS and anthropometric dimensions • Highest correlation of HGS with height, hand span, forearm circumference and hand length dimensions • No significant difference between physical demand levels and HGS for both genders
Moy et al. (2015)	Multistage sampling of households within five randomly selected districts in rural Malaysia *m* = 927 *f* = 1142 aged 30 years and older	Cross-sectional – one point of data collection	To determine the predictors of handgrip strength among adults of a rural community in Malaysia	• Gender • Age • Dominant hand • Height • Weight • BMI	• Jamar Dynamometer (Second handle position) • American Society of Hand Therapists testing procedure • Three measurements on each hand • 15 second rest break between trials • Highest of three measurements chosen for analysis	• Males had higher HGS compared with females (*p* value <0.001) • HGS declined as age increased for both genders (*p* value <0.05) • Those with medical conditions such as diabetes mellitus, hypertension, and high cholesterol had significantly lower HGS (*p* value <0.01) • Positive and significant correlations between HGS and height (*p* value <0.001), weight (*p* value <0.001) and musculoskeletal score (*p* value <0.001) among males only • In the multivariate model for males, age, height, job groups, and diabetes significantly predicted HGS • Dominant HGS significantly higher than the non-dominant hand • Compared with the population in the West, participants had significantly lower HGS
Rostamzadeh et al. (2019)	Convenience sample of 418 (*m* = 220, *f* = 198) office employees aged from 20 to 60 years.	Cross-sectional – one point of data collection	To establish GS norms of Iranian office workers stratified by gender, age-group and hand dominancy. To review the correlation between GS and different demographic and anthropometric variables To investigate the predictors of GS among the study population and to develop the appropriate predictive equations.	• Age • Gender • Dominant hand – identified as which hand they write with • Height • Weight • BMI • Hand length • Palm width • Palm length • Forearm length • Wrist circumference • Forearm circumference • (all measured as per NASA anthropometric source book)	• Jamar Dynamometer (Second handle position) • American Society of Hand Therapists testing procedure • Average of three measurements • 1 minute rest breaks between trials	• There was a significant correlation between HGS and all measured variables, except BMI; suggesting that HGS increases as height, weight, hand length, palm width, palm length, forearm length, wrist circumferences and forearm circumferences of an office worker increase • HGS had the highest correlation (*p* < 0.01) with palm width followed by palm length and hand length, respectively
Rostamzadeh et al. (2020a)	Stratified random sample from a public university in Iran 1740 male workers aged 20–64 years Two occupational groups: light manual workers and office workers	Cross-sectional – one point of data collection	To compare maximum HGS between light manual workers and office employees and investigate if the expected differences are related to anthropometric dimensions of the workers’ forearms and hands.	• Age • Gender • Dominant hand – identified as which hand they write with • Height • Weight • BMI • Hand length • Palm length • Handbreadth • Wrist circumference • Forearm length • Forearm circumference	• Jamar Dynamometer (Second handle position) • American Society of Hand Therapists testing procedure • Average of three measurements • 1 minute rest breaks between trials	• Maximum HGS of light manual workers was significantly higher then office workers for both hands • Dominant HGS was stronger on average than non-dominant for all workers • HGS increased until 35–39 years then gradually decreased for both work groups • Hand breadth and forearm circumference were significantly different between the two groups of workers with light manual workers having greater hand breadth and forearm circumference in both upper limbs • Weight, height and BMI highly correlated with HGS • Hand breadth and forearm circumference highest correlation with HGS for both groups of workers
Rostamzadeh et al. (2020b)	Quai random sample from shopping centres and malls, service centres and public areas to ensure a wide variety of occupational, ethnic and socioeconomic backgrounds. 4282 Iranians (*m* = 2167, *f* = 2115). Two occupational groups: MW and NMW	Cross-sectional – one point of data collection	To create normative data for GS in Iranian healthy population stratified by age, gender and hand side To compare GS of Iranian population with consolidated and international norms To investigate individual predictors of GS among studied demographic and anthropometric parameters To develop prediction equations for GS in Iran.	• Age • Gender • Dominant hand – identified as which hand they write with • Type of work; MW or NMW • Height • Weight • BMI • Hand length • Palm width • Palm length • Forearm length • Wrist circumference • Forearm circumference • (all measured as per NASA anthropometric source book)	• Jamar Dynamometer (Second handle position) • American Society of Hand Therapists testing procedure • Average of three measurements • 1 minute rest breaks between trials	• Hand dominance had a significant effect on HGS (*p* < 0.001). The dominant hand was stronger than non-dominant hand by about 10% and 11% for males and females, respectively • MWs were found to have stronger HGS compared to NMWs, on both sides (*p* < 0.001). Among NMWs, students had a weaker grip than non-students • HGS increases with increasing weight, height, BMI, hand length, palm length, palm width, forearm length, wrist circumference and forearm circumference • Palm width has the highest correlation with HGS in both genders (*p* < 0.01) for dominant and non-dominant hands
Saremi and Rostamzadeh (2019)	Stratified random sampling methods from difference organisations and companies. *m* = 1740 aged from 20 to 64 years 2 occupational groups: LMW tasks (905) and office/clerical workers (835)	Cross-sectional – one point of data collection	To investigate whether light manual workers have higher GS compared to office/clerical employees as non-manual workers To determine anthropometric differences between the two occupational groups. and To determine demographic and anthropometric factors related to GS in each occupational group.	• Age • Gender • Dominant hand – identified as which hand they write with • Work category • Height • Weight • BMI • Hand length • Palm width • Palm length • Forearm length • Wrist circumference • Forearm circumference • (all measured as per NASA anthropometric source book)	• Jamar Dynamometer (Second handle position) • American Society of Hand Therapists testing procedure • Three consecutive measurements on each hand • 1 minute rest break between trials	• HGS in LMW significantly higher than office/clerical employees • Palm width and forearm circumference significantly different between the two occupational groups • LMW had greater palm width and forearm circumference than office/clerical employees • All demographic and anthropometric factors significantly correlated with HGS • Palm width most significant correlation with HGS for LMW and office/clerical employees • Forearm circumference also correlated to HGS for dominant and non-dominant hands of LMW, and office/clerical employees
Saremi et al. (2021)	Certified dentists who specialised in one of three specialities of maxillofacial surgery, endodontics or paediatric dentistry Purposive sample of 720 dental specialists *m* = 330 *f* = 390	Cross-sectional – one point of data collection	To investigate the relationship between dentists’ hand functionality and dental speciality, socio-demographic factors and hand-forearm anthropometrics dimensions	• Age • Gender • Dominant hand – identified by classifying the non-dominant hand as the hand which holds the mirror during treatment • Dental speciality • Height • Weight • BMI • Hand length • Palm length • Palm width • Wrist circumference • Forearm circumference • Forearm length • Clinical experience	• Jamar Dynamometer (Second handle position) • American Society of Hand Therapists testing procedure • Three consecutive measurements on each hand • 1 minute rest break between trials	• Average anthropometric measures were higher in males compared to females • Height and weight significantly correlated to HGS • Hand length and forearm were strongly correlated with HGS • Palm dimensions (length and width) both correlated with HGS • Wrist and forearm circumferences were moderately correlated with HGS • Significant effect for gender as well as age with HGS higher in males than females, whatever the age group • Male and female maxillofacial surgeons had higher mean HGS and forearm circumference than the other specialists • Clinical experience was negatively correlated to HGS for all specialties, suggesting HGS decreased with increasing seniority in dental work
Shim et al. (2013)	Convenience sample of patients visiting health institution *m* = 137 *f* = 199 aged 13–77 years	Cross-sectional – one point of data collection	To establish the normal values of grip and pinch strength among the healthy Korean population and to identify any dependent variables affecting grip and pinch strength.	• Age • Gender • Dominant hand • Height • Weight • Hand width • Hand length • Forearm length • Forearm circumference	• Jamar Dynamometer (Second handle position) • American Society of Hand Therapists testing procedure • Three consecutive measurements on each hand • 1 minute rest break between trials	• All mean strength measurements significantly greater in males than in females (*p* value <0.01) • HGS increased into young adulthood and then declined among the geriatric population • Hand dominance had no significant correlation with measured variables, however dominant hand had greater HGS • All metrics but the forearm lengths were found to correlate with male HGS (*r* = 0.4–0.5) • No significant correlations were found between HGS and all metrics for females
Spruit et al. (2013)	*m* = 224,852 *f* = 224,830 white ethnic background aged 39–73 years as part of the United Kingdom biobank prospective epidemiological study	Cross-sectional – one point of data collection	To develop normative values for right and left handgrip strength after stratification for confounders like gender, age, and height. To develop new normative values for handgrip strength, after stratification for sex, age, and height using individuals from the large UK Biobank dataset without chronic conditions.	• Age • Gender • Height	• Jamar Dynamometer (Second handle position) • American Society of Hand Therapists testing procedure • Three consecutive measurements on each hand	• Men were stronger than women (*p* value <0.001) • A weak inverse correlation was found between HGS and age (*p* value <0.01) • A strong positive correlation was found between HGS and height (*p* value <0.01) • Men, younger individuals, and taller individuals had higher HGS compared with women, older individuals, and shorter individuals
Wang et al. (2018)	Community dwelling and non-institutionalised United States residents aged 18–85 years as part of the normative phase of the NIH Toolbox project *m* = 449 *f* = 783	Prospective cohort study	To provide population-based grip strength reference values and equations for United States residents 18–85 years. Normative data will enable comparison of grip strength values in individuals with or without impairments to the reference values and allow clinicians to provide feedback during the rehabilitation process	• Age • Gender • Dominant hand • Height • Weight • BMI • Ethnicity (including language spoken) • Education	• Jamar Dynamometer (Second handle position) • American Society of Hand Therapists testing procedure • Single maximal trial of 3–4 seconds	• HGS differed significantly by sex (men stronger than women), hand dominance (dominant side stronger) and age (younger adults stronger than older adults) (*p* value <0.001) • Stronger correlation between HGS and height (*r* = 0.61) than the correlation between grip strength and BMI ((*p* value <0.045) • Three variables identified (weight, height and aged cubed) for inclusion in reference equations • Participants with a high school diploma or higher degree were stronger than participants who did not finish secondary education

HGS: handgrip strength; LMW: light manual; MW: manual workers; NIH: National Institutes of Health; NMW: non-manual workers.

**Figure 1.**
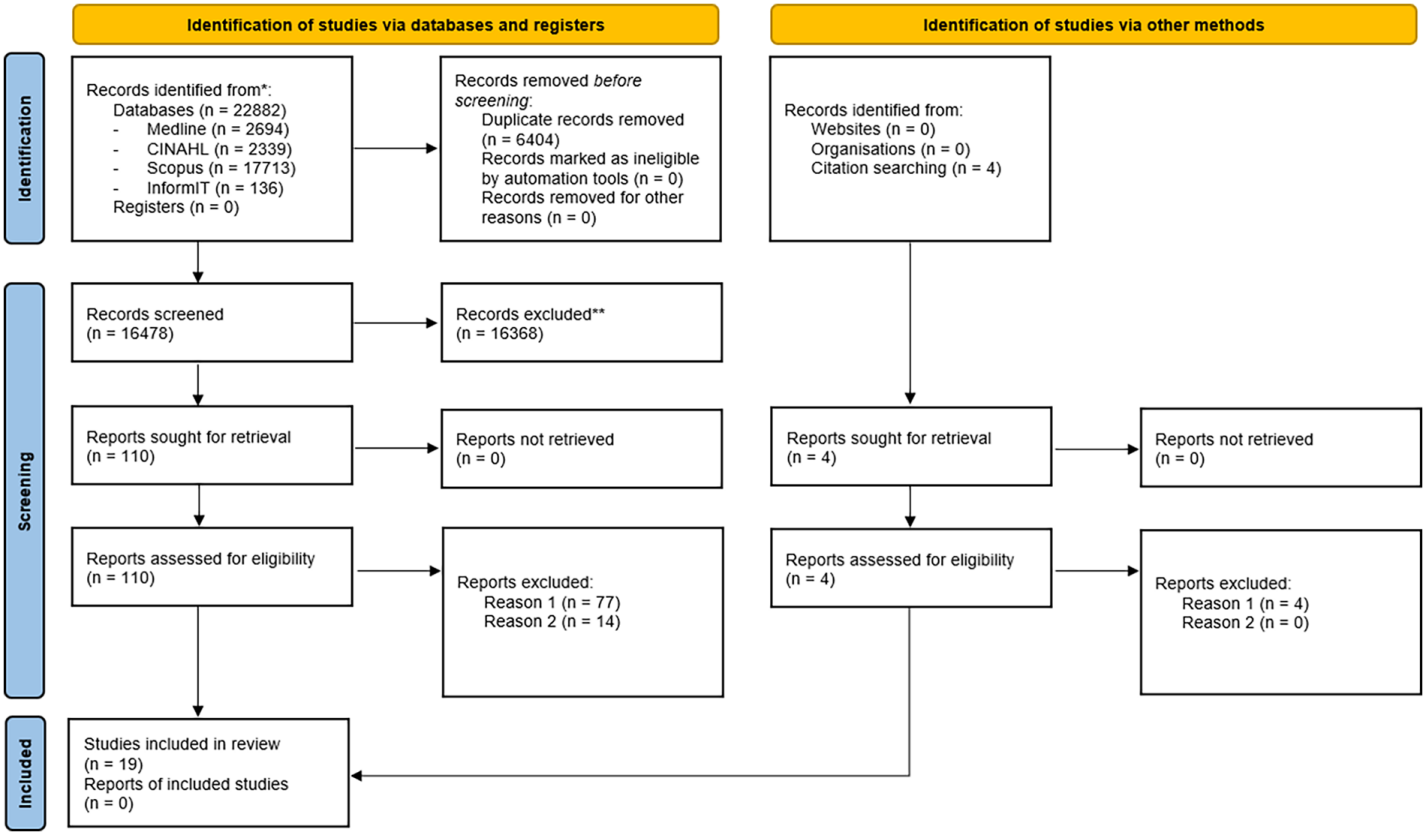
PRISMA flowchart for identification and assessment of eligibility of studies for inclusion in systematic review (Moher et al., 2009). PRISMA: preferred reporting items for systematic reviews and meta-analyses.

The preferred reporting items for systematic reviews and meta-analyses (PRISMA) guidelines were used to guide the selection process as presented in Figure 1. PRISMA flowchart of the literature search process (Page et al., 2021).

## Critical appraisal

Studies were appraised using the McMaster Critical Review Form for Quantitative Studies (Letts, 2007), and data were extracted and synthesised to determine research quality. The author (LM) independently read and scored the included articles on each question, by selecting ‘yes’, ‘no’ or ‘not applicable/stated’ if the item was not relevant to the study. The use of a scoring system allowed for comparison of the results across studies and the evaluation of methodological quality (Alexandratos et al., 2012). As the studies did not involve an intervention protocol, the scoring system was adapted to remove the three points allocated within the intervention criteria; thus, the scores for quality were scaled to a total out of 11 points available. Review of the critical appraisal score was then confirmed by another author (FB). It is important to note that providing a single summary score or scale to identify the research quality can mask deficits in some criteria by scoring high in others (Crowe and Sheppard, 2011). However, the quality of all articles included in the review was considered and identified to be of low to high quality based on the McMaster Critical Appraisal Tool.

## Results

### Study selection

The study selection process followed the PRISMA guidelines and is summarised in Figure 1. Sixteen thousand four hundred and seventy-eight articles were identified from the literature search. After title searching was conducted, 110 articles met the inclusion criteria and full text article reviews were undertaken. Of these, 91 articles were excluded during the evaluation of the full article based on the previously identified inclusion criteria, specifically for variations from the standardised the testing protocol (reason one) and variances in the dynamometer utilised (reason two). The four articles identified during citation searching were also excluded due to variations from the standardised testing protocol. As a result, 19 articles were included in the systematic review and recommendations were based on the results of these studies.

### Study characteristics

Data from all 19 articles was extracted and critiqued and is summarised in Table 1. Ten studies (Angst et al., 2010; Langer et al., 2022; Massy-Westropp et al., 2011; Mohammadian et al., 2015; Rostamzadeh et al., 2019; Rostamzadeh et al., 2020a, 2020b; Saremi and Rostamzadeh, 2019; Saremi et al., 2021; Spruit et al., 2013) were of high quality due to their sample sizes, the variables measured and the methodology employed. Seven studies (Bhat et al., 2021; De Andrade Fernandes et al., 2014; Hatem et al., 2016; Klum et al., 2012; Moy et al., 2015; Shim et al., 2013; Wang et al., 2018) were considered of medium quality, and two (Anjum et al., 2012; Eidson et al., 2017) studies were identified as low quality based on the same analysis.

Sample sizes ranged from smaller convenience samples of 150 participants (Eidson et al., 2017) to larger cohort studies with of 449,000 participants (Spruit et al., 2013). Ten studies had sample populations over 1000 participants (De Andrade Fernandes et al., 2014; Hatem et al., 2016; Massy-Westropp et al., 2011; Mohammadian et al., 2015; Moy et al., 2015; Rostamzadeh et al., 2020a, 2020b; Saremi and Rostamzadeh, 2019; Spruit et al., 2013; Wang et al., 2018). The large sample sizes of these studies provides greater confidence when translating the research findings back to the general population (Banerjee and Chaudhury, 2010).

Seventeen studies used a cross-sectional study design with one point of data collection. The only exceptions to this study design were Wang et al. (2018) who employed a prospective cohort study design drawing data from The United States National Institutes of Health (NIH) Toolbox and Massy-Westropp et al. (2011) who used a cross-sectional study design with two points of data collection being phone interviews and face-to-face HGS assessment. Studies examined Asian populations living in the United Kingdom (Anjum et al., 2012), and Western population studies from Switzerland (Angst et al., 2010), Germany (Klum et al., 2012), Israel (Langer et al., 2022), the United Kingdom (Spruit et al., 2013), Australia (Massy-Westropp et al., 2011) and the United States (Eidson et al., 2017; Wang et al., 2018). Studies were also conducted in Brazil (De Andrade Fernandes et al., 2014), Iran (Mohammadian et al., 2015; Rostamzadeh et al., 2019; Rostamzadeh et al., 2020a, 2020b; Saremi and Rostamzadeh, 2019; Saremi et al., 2021), Korea (Shim et al., 2013), Malaysia (Moy et al., 2015) and Egypt (Hatem et al., 2016). The study by Bhat et al. (2021) sought to evaluate HGS and hand anthropometry for young adults based on gender and eight varied ethnicities.

When examining the factors that may influence HGS, six common factors were identified: gender, age, hand dominance, ethnicity, occupation and anthropometric characteristics. All studies examined HGS in relation to age and gender. The inclusion of occupation/occupational strain or physical demand levels was only discussed in seven studies with the majority of studies finding a positive correlation between occupation/physical demand and HGS (Angst et al., 2010; Moy et al., 2015; Rostamzadeh et al., 2019; Rostamzadeh et al., 2020a, 2020b; Saremi and Rostamzadeh, 2019). Angst et al. (2010) concluded that the occupational demand on the hand may have caused bias in relation to the HGS scores. In contrast. Klum et al. (2012) found no significant correlation between occupational manual strain and HGS.

Five studies (Bhat et al., 2021; Langer et al., 2022; Mohammadian et al., 2015; Moy et al., 2015; Rostamzadeh et al., 2020b) investigated the influence of ethnicity on HGS by comparing the HGS results of specific ethnic groups to other population sets. These studies all concluded that populations from developed countries or norms derived from predominantly Caucasian populations had increased HGS results compared with South Asian and African populations.

The anthropometric characteristics of height, weight and the resultant BMI were explored in relation to HGS in numerous studies with conflicting results. Height rather than BMI was shown to have the strongest positive correlation with HGS (Angst et al., 2010; Hatem et al., 2016; Mohammadian et al., 2015; Moy et al., 2015; Saremi et al., 2021; Spruit et al., 2013; Wang et al., 2018). Various other anthropometric characteristics including hand length, forearm length, hand width/palm width and forearm circumference were also analysed in comparison with HGS. Of these other anthropometric characteristics examined, hand length and hand width/palm width were found to have the strongest positive correlation with HGS (Eidson et al., 2017; Mohammadian et al., 2015; Rostamzadeh et al., 2019; Rostamzadeh et al., 2020a, 2020b; Saremi and Rostamzadeh, 2019).

## Discussion

The aim of this review was to identify the influence of various biological and functional factors for working adult HGS, including which factors most strongly predict HGS. The major influencing factors identified were age and gender. This finding is consistent with previous research which has concluded that there is a well-established relationship between gender and HGS, and age and HGS (Agnew and Maas, 1982; Dodds et al., 2016; Mathiowetz et al., 1985).

Currently, most HGS normative data sets are classified by age and gender only. It is well documented that grip strength declines with increasing age (Agnew and Maas, 1982; Mathiowetz et al., 1985). De Andrade Fernandes et al. (2014) found a curvilinear relationship with HGS peaking during the third decade, followed by a decrease as age progresses. Hatem et al. (2016), Moy et al. (2015), Mohammadian et al. (2015), Saremi et al. (2021) and Shim et al. (2013) determined that a significant inverse correlation exists between age and HGS for both genders of the working population. This decline in HGS may be considered part of the normal ageing process that sees a decline in muscle mass and a likely consequent reduction in muscular strength forces during HGS testing.

It is also widely accepted that HGS among men is higher than the HGS of women. Several studies in this review supported this viewpoint. Results from the studies by Langer et al. (2022), Moy et al. (2015), Shim et al. (2013) and Spruit et al. (2013) concluded that all strength measurements were significantly greater in men than in women. Men are known to have higher percentages of muscle mass compared to women, which may explain why the variation in HGS exists between genders. Klum et al. (2012) concluded that gender was the most important factor when predicting HGS. Recent studies have identified other biological and functional factors which should be considered in addition to age and gender in order to improve the interpretation and evaluation of an individual’s HGS (Anjum et al., 2012; Dodds et al., 2016; Klum et al., 2012; Leong et al., 2016; Mohammadian et al., 2015; Rostamzadeh et al., 2019b, 2020a; Saremi and Rostamzadeh, 2019; Saremi et al., 2021).

### Biological factors

Biological factors found to be relevant to HGS include anthropometric measures such as height, weight, BMI, various hand and forearm measurements and ethnicity. The anthropometric factors found to have the strongest correlation with HGS were height, hand length and hand width/palm width (Angst et al., 2010; Eidson et al., 2017; Hatem et al., 2016; Klum et al., 2012; Mohammadian et al., 2015; Moy et al., 2015; Rostamzadeh et al., 2019b, 2020a; Saremi and Rostamzadeh, 2019; Saremi et al., 2021; Spruit et al., 2013; Wang et al., 2018). Height alone was determined to have the most significant correlation with HGS in the findings from Mohammadian et al. (2015), Angst et al. (2010), Moy et al. (2015), Spruit et al. (2013) and Wang et al. (2018). The correlation between height and HGS within these studies is important to consider given the quality of these studies. These studies were scored as high and medium quality during the critiquing process due to their sample sizes, variables measured and the methodology utilised. Previous research (Agnihotri et al., 2008) concluded that a person’s hand length is a prime criterion to estimate height and this in combination with hand width may provide a participant with a mechanical advantage when squeezing the dynamometer during HGS testing, particularly when using the standardised second handle position. The findings from this review support these previous findings.

Height and weight were identified as having a signification correlation to HGS in the studies undertaken by Angst et al. (2010), Anjum et al. (2012), Hatem et al. (2016) and Rostamzadeh et al. (2019). Interestingly, BMI, which is the relationship between height and weight, was not found to have a relationship with HGS in the studies by Anjum et al. (2012) and S. Rostamzadeh et al. (2019). The current review found that BMI did not correlate to HGS for Asian, Middle Eastern and European populations (Anjum et al., 2012; Rostamzadeh et al., 2019) or those with higher BMIs (Massy-Westropp et al., 2011) where the relationship was reduced or even reversed. In the study by Massy-Westropp et al. (2011), only 27 participants were assessed to have a low BMI which limited the ability to investigate the relationship between HGS and low BMI. When evaluating BMI, it is commonly accepted that BMI correlates strongly with weight but is independent of height (Sperrin et al., 2016). Therefore, anthropometric characteristics of height and hand length and hand width/palm width are not dependent on body weight, and this may explain why BMI does not always have a positive correlation to HGS.

Of the hand measurements taken and compared to HGS, hand width/palm width and forearm circumference provided the strongest relationship. The only other anthropometric measurement seen to correlate positively with HGS was hand length. This relationship may link to the strong correlations seen between height and HGS. As previously discussed, increased height generally results in increased limb lengths for an individual. Saremi and Rostamzadeh (2019) hypothesised that individuals with larger hands may have increased HGS due to their greater muscle mass. Similarly findings from Bhat et al. (2021) suggested that men had larger anthropometric measurements (around 10%–-15% greater) compared to women which may also assist in explaining the strong correlation between HGS and gender.

Body composition such as height, weight, limb length and skeletal muscle mass may vary among population groups of different ethnic backgrounds (De Andrade Fernandes et al., 2014). Anjum et al. (2012) concluded that Asian populations were found to have lower HGS compared to European populations. Bhat et al. (2021) concluded that average HGS varies among differing ethnic groups and this variance may correlate to anthropometric measurements such as height and hand size which are influenced by ethnicity. This supports the need to ensure population specific normative values are being utilised for comparison amongst population groups. Wang et al. (2018) discussed that although there are numerous peer reviewed studies that provide HGS normative values for populations outside of the United States, with most based on small convenience samples. Future research should be aimed at developing population specific norms.

### Functional factors

The relationship between HGS and hand dominance has shown that typically the dominant hand is stronger; however, this correlation is weaker for left hand dominant participants (Bohannon, 2003). This was supported by Moy et al. (2015), S. Rostamzadeh et al. (2019) and Shim et al. (2013) who found dominant HGS to be significantly greater than non-dominant HGS regardless of gender. Hand dominance was recorded in a number of studies; however, due to low rates of left hand dominant participants, normative values were not categorised into dominant and non-dominant groups. When considering hand dominance and various population groups, De Andrade Fernandes et al. (2014) determined the strength difference between hands was found to be consistent, regardless of ethnicity. As there is a documented difference between dominant and non-dominant HGS, using categories identifying right or left hand dominance would aid in improved interpretation of the HGS normative data.

The studies by Angst et al. (2010), Klum et al. (2012) and Mohammadian et al. (2015) found occupation/varying physical demand levels did not have a high predictive power for HGS. These studies were carried out with vastly different population samples of European and Iranian workers whose occupation and the physical work demands required are likely to vary significantly. The study by Moy et al. (2015) based on a Malaysian population found males who performed heavy manual work had higher HGS compared to those who performed light work, however the type of occupation did not predict HGS for females. Moy et al. (2015) hypothesised that this inconsistency for occupation to predict HGS for both genders may be due to the decreased diversity in occupations for females and the small proportion of females who were currently employed or had ever worked. Body size and composition contributes to an individual’s physical capabilities and as such may have an indirect correlation to job performance (Roberts et al., 2016). The occupations performed by females are generally less physically demanding than males who have increased musculature compared to females. Several studies on the Iranian population all found significant correlation between HGS and occupation (Rostamzadeh et al., 2019, 2020a, 2020b; Saremi and Rostamzadeh, 2019; Saremi et al., 2021). These studies either only focused on one type of occupation such as office-based workers (Rostamzadeh at al., 2019) and dentists (Saremi et al., 2021) or divided workers into two categories: manual workers and non-manual workers (Rostamzadeh et al., 2020a, 2020b; Saremi and Rostamzadeh, 2019). Having broad occupational categories which were distinct from one another may have aided in demonstrating the correlation between HGS and occupation.

Unskilled manual occupations are often performed by workers from lower socio-economic backgrounds. Leong et al. (2016) discussed variations in muscle strength are linked to differences in socio-economic status and education levels. Possible variations in muscle strength and HGS may be due to dietary differences between the various populations due to the differences in socio-economic status (Leong et al., 2016). Wang et al. (2018) also concluded that participants with higher education levels were not stronger than participants who did not finish secondary education. Large variations of physical demand levels are required to perform the diversity of occupations within different cultures. Cultural differences also influence the types of occupations performed, socio-economic status and education of individuals. Therefore, further investigation into the significance of occupation in relation to HGS is required.

### Implications for practice

This study identified significant variation in testing methodologies across studies examining HGS in conjunction with biological and functional factors. HGS testing is commonly used by occupational therapists as a standard measure of hand function, and it is suggested when analysing HGS select biological and functional factors beyond age and gender are considered to improve HGS evaluation. By considering the influence of these select biological and functional factors, occupational therapists can use their occupational knowledge within the evaluation of HGS and combine this perspective with the biomedical element of the quantifiable HGS scores for an improved understanding of how HGS scores relate to an individual’s everyday tasks.

### Limitations and future research

While this study examined various research studies examining the influence of various biological and functional factors on HGS, some limitations were evident. Due to the volume of research available on HGS, included studies were limited to 2010–-2023 to ensure the most recent research studies were captured. Additionally, several studies offering detailed examining of numerous biological and functional factors in relation to HGS were excluded from inclusion in the review due to non-use of the complete ASHT HGS testing protocol. Several research studies included within the review were completed by the same research group and based on a specific ethnic population. Due to the variability in body dimension within various ethnic populations, this may affect the generalisability of these study results to a global population. Future research on HGS should ensure the study design considers the use of a standardised methodology when assessing HGS to ensure valid and reliable results. Additionally, adoption of a consistent testing protocol would facilitate comparison of study findings across research studies.

## Conclusion

Various biological and functional factors have been examined in relation to adult’s HGS with the aim of developing an improved understanding of how to interpret and compare HGS results with normative data sets. This research found that in addition to the accepted factors of age and gender, other biological and functional factors influence HGS of working adults. It is also critical to ensure comparison is made between the same populations when comparing individuals to HGS normative data sets. When analysing HGS, it is recommended that occupational therapists and other health professionals consider more factors than age and gender to provide increased contextualisation and improved confidence when guiding decision-making for treatment and rehabilitation of the hand. This review has identified height as the most significant factor in correlation to HGS along with the additional anthropometric factors of hand length and hand width/palm width. These anthropometric factors also link to ethnicity as populations from different geographical locations can have varying body sizes. When developing new normative data sets for HGS, anthropometric characteristics such as height, weight, hand length and hand width/palm width, hand dominance and occupation should be considered along with the established categories of age and gender to allow for improved evaluation of an individual’s HGS. When assessing and evaluating HGS, all health professionals regardless of discipline need to consider the influence of these biological and functional factors in addition to age and gender for increased contextualisation of the HGS results in relation to an individuals’ body size and daily occupations.

**Key findings**

- Height followed by hand width/palm width most strongly correlated to HGS
- It is critical to ensure comparison is made between the same populations when comparing individuals to HGS normative data sets
- Consideration of select biological and functional factors provides contextualisation to guide decision making for treatment and rehabilitation

**What the study has added**

This study identified significant variation in testing methodologies across studies examining HGS in conjunction with biological and functional factors. When analysing HGS, it is suggested that factors beyond age and gender are considered to improve HGS evaluation.
